# Diverse molecular functions of m^6^A mRNA modification in cancer

**DOI:** 10.1038/s12276-020-0432-y

**Published:** 2020-05-13

**Authors:** Seung Hun Han, Junho Choe

**Affiliations:** 1 Department of Life Science, College of Natural Sciences, Hanyang University, Seoul, 04763 Republic of Korea; 2 Research Institute for Natural Sciences, Hanyang University, Seoul, 04763 Republic of Korea; 3 Research Institute for Convergence of Basic Sciences, Hanyang University, Seoul, 04763 Republic of Korea

**Keywords:** RNA metabolism, Translation, Transcriptomics, Cancer

## Abstract

*N*^6^-methyladenosine (m^6^A), the most prevalent chemical modification found on eukaryotic mRNA, is associated with almost all stages of mRNA metabolism and influences various human diseases. Recent research has implicated the aberrant regulation of m^6^A mRNA modification in many human cancers. An increasing number of studies have revealed that dysregulation of m^6^A-containing gene expression via the abnormal expression of m^6^A methyltransferases, demethylases, or reader proteins is closely associated with tumorigenicity. Notably, the molecular functions and cellular consequences of m^6^A mRNA modification often show opposite results depending on the degree of m^6^A modification in specific mRNA. In this review, we highlight the current progress on the underlying mechanisms of m^6^A modification in mRNA metabolism, particularly the functions of m^6^A writers, erasers, and readers in the context of tumorigenesis.

## Cancer: monitoring messenger RNA modifications

A thorough investigation into the role and function of RNA modifications in cancers could yield novel therapies. The chemical modification of messenger RNA (mRNA), the molecule that carries code from DNA to synthesize proteins, is thought to play a role in influencing genetically inherited traits and diseases. A common modification found in mRNA is *N*^6^-methyladenosine (m^6^A). Disruption to the regulation of m^6^A modification has been linked with human cancers. Junho Choe and Seung Hun Han at Hanyang University in Seoul, South Korea, reviewed current understanding of the molecular mechanisms behind m^6^A modification, with particular reference to tumor formation. The researchers point out that abnormal expression of proteins associated with m^6^A may lead to heightened expression of cancer-related genes. More extensive m^6^A modification levels are also linked to tumor formation.

## Introduction

Since the discovery of the DNA double-helix structure in the 1950s, how genetic information is controlled and inherited has been a fundamental question. The discovery that alteration of the chromatin structure and DNA modifications affect heritable phenotypes in addition to the DNA sequence itself opened up a new field of epigenetics^1^. Similarly, many recent studies have proposed various chemical modifications of RNA as another layer of post-transcriptional gene expression regulation termed “epitranscriptomics”^2–4^. Post-transcriptional regulation is critical for the control of gene expression programs that dictate a variety of cellular functions and cell fate decisions. To date, at least 160 different chemical modifications have been identified in multiple RNA species, including messenger RNAs (mRNAs), transfer RNAs (tRNAs), ribosomal RNAs (rRNAs), noncoding RNAs (ncRNAs), and viral RNA genomes^4,5^. Although the majority of these modifications map to noncoding RNAs, increasing evidence implicates multiple mRNA modifications as components of another layer of gene expression regulation^2,6^.

Discovered in the 1970s, *N*6-methyladenosine (m^6^A) is the best-characterized RNA modification and particularly is involved in almost all stages of the mRNA life cycle, including splicing, export, translation, and stability^7–11^. It is the most prevalent mRNA modification, with approximately one-fourth of the eukaryotic mRNAs harboring at least one m^6^A-modified base^3,12^. The m^6^A modification is found in multiple organisms and associated with various cell functions, including meiosis in yeast^13,14^, plant development^15^, mouse spermatogenesis^16^, mouse embryogenesis^17^, and various cancers^18–22^.

In this review, we discuss the current understanding of m^6^A mRNA modification regulation at the molecular level and its various cellular effects. In particular, we highlight the emerging understanding of m^6^A mRNA modification in cancer.

## Mechanism of dynamic m^6^A modification

**Fig. 1:**
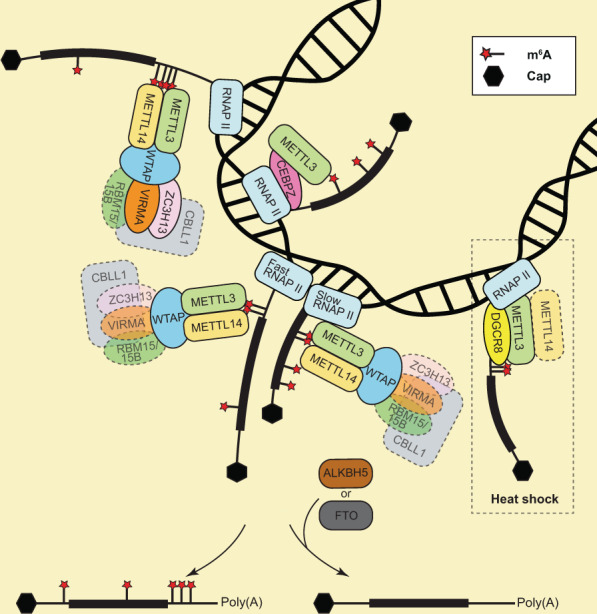
An overview of cotranscriptional m^6^A mRNA modification. Introduction of m^6^A modification is currently suggested to occur cotranscriptionally in the nucleus. Individually transcribing mRNAs illustrate the different modes of cotranscriptional m^6^A modification, including different compositions of associated DNA- and RNA-binding proteins with distinct methylation sites. The thick line represents the coding sequence, and the thin line represents the UTR. The dashed box indicates the heat shock condition.

The discovery of methyltransferases (also known as m^6^A writers) and demethylases (also known as m^6^A erasers) provided evidence that m^6^A modification is a dynamic and reversible event^23^. In addition to the combined action of m^6^A writers and erasers on m^6^A modification regulation, m^6^A reader proteins contribute to the regulation of the fate of m^6^A-containing RNAs. The m^6^A modification is the methylation of the sixth position of nitrogen atom of adenosine, with the cellular methyltransferase substrate *S*‑adenosylmethionine serving as the methyl donor for m^6^A formation^24,25^. Methyltransferase-like protein 3 (METTL3, also known as MT-A70) and METTL14 form a heterodimer at a ratio of 1:1, and they functions as a catalytic core complex recognizing the DRACH motif (D = A, G, or U; R = G or A; and H = A, C, or U) and inducing m^6^A modification of mRNA^12,24^. Growing evidence has revealed that METTL3 plays a central role in introducing m^6^A onto nascent transcripts cotranscriptionally, while METTL14 supports binding of the METTL3 protein to the target mRNA (Fig. 1)^26,27^. In addition, at least five other proteins are involved in the regulation of m^6^A mRNA modification, although it often shows a slightly different composition of the protein complex in each study. While they lack methyltransferase activity, they stabilize the METTL3/14 complex and facilitate its localization to the specific RNA sites for m^6^A modification^28,29^. Wilms tumor 1-associated protein [WTAP, also known as female-lethal(2)d] recruits other proteins to the METTL3/14 complex, thereby affecting the overall levels of m^6^A modification^30^. RNA-binding motif 15 (RBM15) protein and its paralog RBM15B have been shown to interact with METTL3 in a WTAP-dependent manner^28,31^. It has been suggested that they preferentially bind to U-rich regions in RNA and recruit the METTL3/14-WTAP complex to sites proximal to the m^6^A consensus motifs^31^. Vir-like m^6^A methyltransferase associated protein (VIRMA, also known as Virilizer or KIAA1429) was recently found to mediate mRNA methylation near the stop codon in the 3′ untranslated region (UTR), where it plays a role in alternative polyadenylation^32^. In mouse embryonic stem cells (mESCs), Cbl proto-oncogene like 1 (CBLL1, also known as Hakai) protein and zinc finger CCCH-type containing 13 (ZC3H13) proteins have been shown to be required for the nuclear localization of ZC3H13-WTAP-VIRMA-CBLL1, which promotes m^6^A mRNA modification^29^. In *Drosophila*, ZC3H13 has also been shown to act as an adapter protein between WTAP and RBM15 in the methyltransferase complex to support efficient methylation^28^. On the other hand, methyltransferase-like protein 16 (METTL16) was recently found to be critical for m^6^A modification in several pre-mRNAs, U6 small nuclear RNAs (U6 snRNAs), and noncoding RNAs containing a specific stem-loop structure^33–35^. Interestingly, METTL16 has been shown to control *S*-adenosylmethionine levels by regulating the expression of a *S*-adenosylmethionine synthetase methionine adenosyltransferase 2 A (MAT2A) by the enhanced splicing of a retained intron^33,34^. When METTL16 is depleted, the level of m^6^A in a cell decreases by ~20%^33^.

To date, two mammalian m^6^A demethylating enzymes have been identified, namely, the fat mass and obesity-associated protein (FTO) and a-ketoglutarate-dependent dioxygenase alk B homolog 5 (ALKBH5) protein^36,37^. FTO was the first identified m^6^A demethylase originally found to be associated with increased body mass and obesity in humans^36,38^. Demethylation of m^6^A by FTO generates an intermediate product, *N*6-hydroxymethyladenosine (hm^6^A), which is then further oxidized to *N*6-formyladenosine (f^6^A)^39^. However, the potential functions of these intermediate products remain unclear. While several studies have provided evidence that depletion of FTO increases the level of total m^6^A, another recent report suggested FTO preferentially demethylates 2′-*O*-dimethyladenosine (m^6^Am), which is found adjacent to the 7-methylguanosine (m^7^G) cap in mRNA, thereby influencing mRNA stability^40^. Most recently, FTO was also shown to demethylate *N*1-methyladenosine (m^1^A) in tRNAs^41^. ALKBH5, the second identified m^6^A demethylase, preferentially recognizes the m^6^A mark for demethylation in a consensus sequence-dependent manner; thus, it is considered as a better candidate for global m^6^A demethylation^37^.

## Molecular functions of m^6^A in mRNA metabolism

**Fig. 2:**
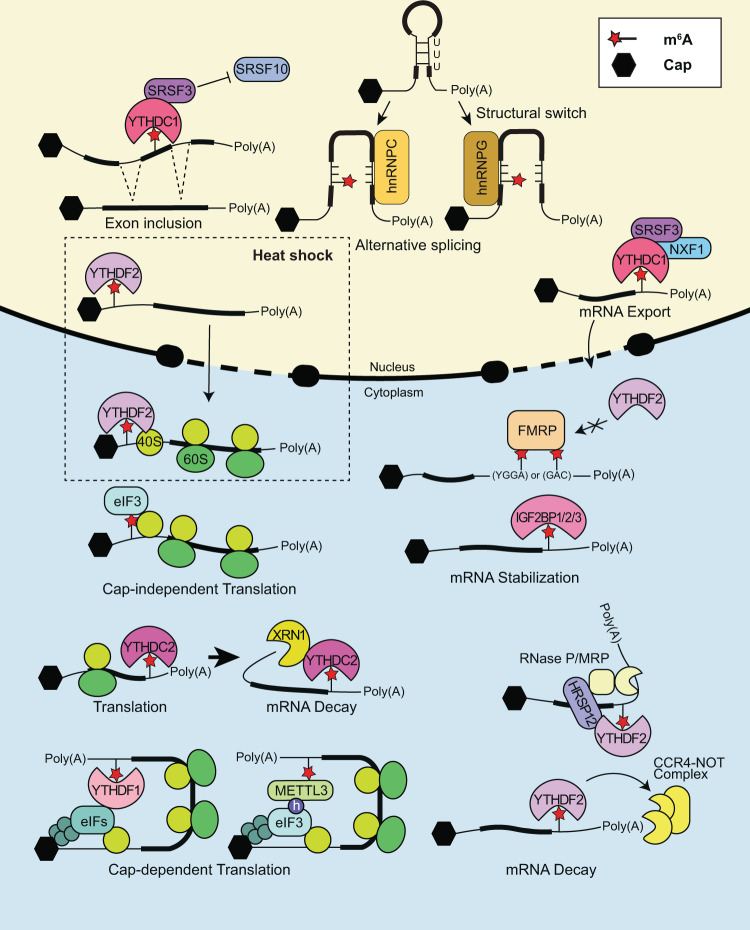
Molecular details for m^6^A-mediated mRNA metabolism. Multiple m^6^A reader proteins dynamically regulate m^6^A-containing mRNA metabolism, including alternative splicing, mRNA export, structural switch, translation, and mRNA stability, depending on the specific m^6^A-bound reader protein. The thick line represents the coding sequence, and the thin line represents the UTR. The dashed box indicates the heat shock condition.

Gene expression is the result of orchestrated transcriptional and post-transcriptional regulation. Recently, an increasing number of studies have suggested m^6^A mRNA modification as a layer of gene expression regulation previously unrecognized. Various m^6^A reader proteins are involved in many processes of overall mRNA metabolism (Fig. 2).

### Cotranscriptional m^6^A modification

In general, m^6^A modifications of mRNAs are enriched near translation stop codons in the 3′ UTR^3,12,42^. However, this characteristic varies among different mRNAs and depends on the tissue. There are several lines of evidence indicating that the m^6^A modification is a cotranscriptional event (Fig. 1)^18,26,27^. One report showed that METTL3 binds to chromatin in a transcription-dependent manner and cotranscriptionally methylates nascent transcripts^26^. In a case of acute myeloid leukemia (AML), METTL3 can be recruited to the promoter region independent of METTL14 by binding to CCAAT/enhancer-binding protein zeta (CEBPZ)^18^. METTL3 can induce m^6^A modification cotranscriptionally within the coding region of the associated transcripts, ultimately resulting in translation enhancement^18^. Moreover, it has been shown that cotranscriptional modification of m^6^A is dependent on the activity of RNA polymerase II (RNAP II)^27^. A low rate of transcriptional activity induces increased levels of m^6^A modification throughout the gene body, resulting in reduced levels of translation^27^. On the other hand, in the case of heat shock stress, METTL3 can be recruited with DGCR8 to the chromatin of heat shock responsive genes in the region of the transcription ending site, where it subsequently methylates nascent mRNAs, leading to the degradation of the target mRNAs as a consequence^26^. Considering the accumulating evidence that the m^6^A modification is mainly found around the translation stop codon in mRNAs^3,12,42^ and that VIRMA preferentially mediates mRNA methylation near the stop codon in the 3′ UTR^32^, further studies are required to clarify such discrepancies in the methylation mechanism. Moreover, despite consistent results showing that m^6^A modification is a cotranscriptional event, the molecular consequences of this modification vary among different studies. Therefore, further research is required to determine the regulating factors that lead to these discrepancies.

### m^6^A promotes alternative splicing

Multiple model organism studies have shown that dynamic m^6^A modification alters mRNA splicing. In *Drosophila*, mutation of IME4 (a METTL3 homolog) influences sex determination by modulating female-specific splicing of the Sex-lethal (Sxl) gene^43,44^. In addition, the *Drosophila* orthologs of VIRMA and/or ZC3H13 have been shown to regulate alternative splicing of pre-mRNAs involved in sex determination^28^. m^6^A demethylases were also reported to be involved in splicing machinery^37,45,46^. FTO regulates mouse pre-adipocyte differentiation by regulating the alternative splicing of the genes involved in adipogenesis^45^. ALKBH5 regulates splicing by removing m^6^A from pre-mRNAs and allows the production of a subset of mRNAs containing relatively long 3′ UTRs in mouse germ cells^37,46^. While m^6^A writers and erasers regulate alternative splicing by modulating the levels of m^6^A modification, m^6^A reader proteins directly regulate splicing^8,47^. The m^6^A-bound YTHDC1 associating with splicing factor SRSF3 has been shown to block the binding of SRSF10 to m^6^A-modified RNA, promoting exon inclusion in the selected transcripts^8,48^. Moreover, m^6^A modification influences mRNA structural changes, which allows heterogeneous nuclear ribonucleoprotein C (hnRNPC) and hnRNPG binding^9,47^. While hnRNPC binds opposite strand U-rich sequences after the disruption of RNA base pairing by m^6^A modification^9^, hnRNPG preferentially binds to purine-rich motifs, including m^6^A sites^47^. Binding of either hnRNPC or hnRNPG influences the alternative splicing of m^6^A-modified transcripts^9,47^. Finally, METTL16 induces the m^6^A modification of U6 snRNA, which base pairs with 5′ splice sites of pre-mRNAs during splicing, suggesting that METTL16 plays an important role in mRNA splicing^34,35^.

### m^6^A facilitates mRNA export

mRNA export is also influenced by m^6^A modification. ALKBH5-deficient cells exhibit increased levels of cytoplasmic m^6^A-containing mRNA, suggesting that the m^6^A modification accelerates mRNA export^37^. Another report showed that YTHDC1 facilitates the export of m^6^A-modified mRNAs via its interaction with nuclear RNA export factor 1 (NXF1)^10^.

### m^6^A alters RNA structure

It has been well established that gene expression is largely affected by the secondary and tertiary structures of mRNA^49^. Introduction of m^6^A modification promotes the destabilization of A/U pairings, resulting in alterations to the thermostability of RNA duplexes and changes in the RNA secondary structure^50^. Another study demonstrated that RNA structural changes caused by the introduction of m^6^A also alter the interaction between RNAs and proteins^9,47^.

### m^6^A regulates translation efficiency

Many m^6^A reader proteins are reported to be crucial for the efficient translation of methylated mRNAs. Members of the YT521-B homology (YTH) domain-containing protein family have been identified as direct m^6^A readers, including YTHDF1, YTHDF2, YTHDF3, YTHDC1, and YTHDC2^7,11,51–56^. Among these proteins, YTHDF1, YTHDF3, and YTHDC2 have been shown to promote target mRNA translation^11,51–53^. YTHDF1 selectively binds to m^6^A sites near the stop codon and cooperates with translation initiation factors to promote the translation of the target mRNAs^51^. YTHDF3 cooperates with YTHDF1 in the regulation of translation by interacting with a common set of ribosomal proteins^52^. YTHDC2 has been suggested to play a role in enhanced translation levels while reducing target mRNA abundance^53^. Furthermore, increased levels of YTHDF2 translocate to the nucleus under heat shock stress and bind m^6^A in the 5′ UTR of a subset of stress-induced mRNAs, protecting them from FTO-mediated demethylation and promoting their cap-independent translation^57^. Eukaryotic translation initiation factor 3 (eIF3) is also considered an m^6^A-binding protein. mRNAs containing m^6^A modification in the 5′ UTR can be recognized by direct binding of eIF3 to the methylated region, which in turn recruits the 43 S complex to initiate translation in a cap-independent manner in the absence of the cap-binding protein eIF4E^58^. However, the mechanism of eIF3 in the recognition of m^6^A is not yet clearly understood. Interestingly, most recent studies have suggested that the m^6^A writer protein METTL3 also functions as a reader protein in the cytoplasm, promoting the translation of a large subset of target mRNAs^21,22^. These studies revealed that 3′ UTR m^6^A modification near the stop codon significantly increases translation through mRNA looping, governed by the interaction between METTL3 at the 3′ UTR and the translation initiation factor eIF3 subunit h (eIF3h) at the 5′ end^21,22^.

### m^6^A regulates mRNA stability

An increasing number of studies have demonstrated that m^6^A modification influences mRNA stability. Various structural and functional studies suggest that all three YTHDF reader proteins (YTHDF1, YTHDF2, and YTHDF3) may share the same subset of target mRNAs^51,52^. However, accumulating evidence suggests that YTHDF2 is the major factor involved in the degradation of m^6^A-containing mRNA either through exoribonucleolytic decay or the endoribonucleolytic cleavage pathway^55,56^. YTHDF2 has been shown to selectively recognize m^6^A sites and recruit the CCR4-NOT deadenylase complex directly, which in turn recruits exosomes (3′-to-5′ exoribonuclease) to initiate mRNA decay^56^. Other recent studies revealed that YTHDF2 promotes the translocation of m^6^A-containing mRNA from the translation machinery to processing bodies (P bodies), where cellular proteins participating in mRNA degradation are enriched^7,59^. In addition, a very recent study revealed the YTHDF2-mediated endoribonucleolytic cleavage of m^6^A-containing mRNAs^55^. Mechanistically, heat-responsive protein 12 (HRSP12, also known as reactive intermediate imine deaminase A homolog, UK114 antigen homolog, and 14.5 kDa translational inhibitor protein) bridges m^6^A-bound YTHDF2 to an endoribonuclease, RNase P/MRP, triggering the endoribonucleolytic cleavage of an m^6^A-containing mRNA^55^. Another study suggested that YTHDC2 recruits the 5′ to 3′ exoribonuclease XRN1 for subsequent m^6^A-containing mRNA degradation^54^. In addition to the YTH proteins, a variety of other RNA-binding proteins are involved in the regulation of m^6^A-containing mRNA stability. Fragile X mental retardation protein (FMRP) can bind to the sequence motifs YGGA (Y = C or U) and GAC, which likely overlap with the DRACH motif involved in m^6^A modification, resulting in stabilization of the m^6^A-containing mRNA through the competition of FMRP with YTHDF2^60^. In another case, stress granule protein (G3BP1) has binding affinity for m^6^A-methylated transcripts, promoting their demethylation and resulting in stabilization of the target mRNAs^61^. Insulin-like growth factor 2 mRNA-binding protein (IGF2BP) 1, 2, and 3 or human antigen R (HuR, also known as ELAVL1) have also been reported to stabilize m^6^A-containing mRNAs^62,63^.

## Molecular functions of m^6^A in various cancers

**Fig. 3:**
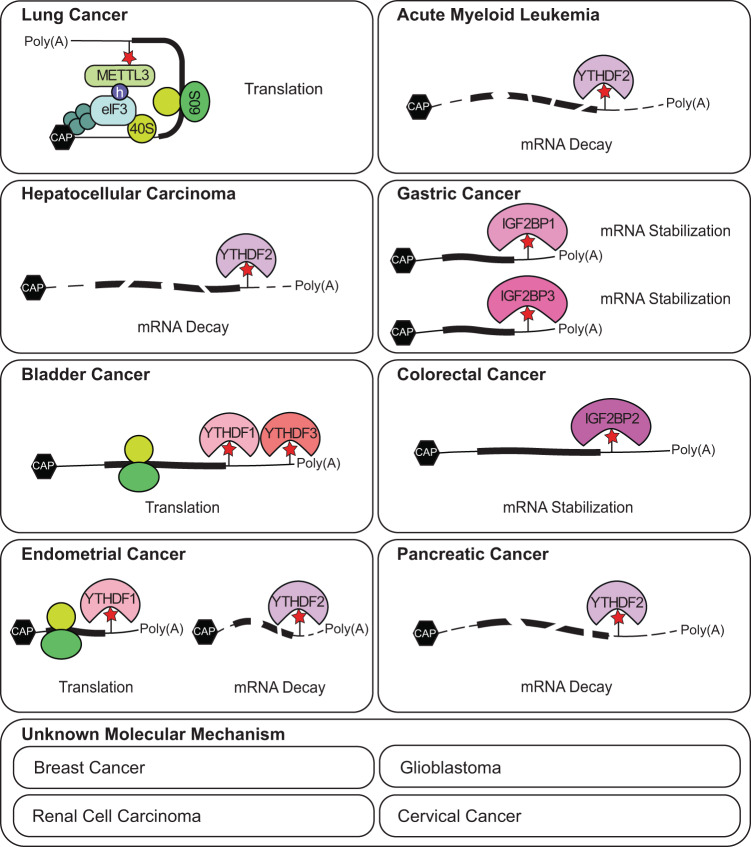
m^6^A-mediated mRNA regulation in tumorigenesis. A number of studies have identified the molecular mechanism of m^6^A-mediated mRNA regulation and their effects on tumorigenesis. To date, m^6^A-mediated regulation of translation or mRNA stability has been demonstrated, while the relevance of pre-mRNA splicing or mRNA export remains unclear for specific cancer types.

Interest in m^6^A modification has been extended to many human diseases as well as to its molecular function. In particular, an increasing number of studies are examining the role of m^6^A-mediated gene expression regulation in cancers. In general, many different signaling pathways converge onto translation machinery to satisfy the increased anabolic demands of cancers. Given the crucial function of m^6^A modification in regulating mRNA metabolism, it is reasonable to speculate that m^6^A modification plays an important role in human carcinogenesis. Nonetheless, the molecular details of how m^6^A modification affects the cellular phenotype of cancer are still being investigated. The physiological effects of m^6^A mRNA modification in cancer often lead to opposite results (Table 1); thus, further understanding of a balanced m^6^A modification is required for the treatment of cancer. Here, we highlight recent insights into the biological functions of m^6^A mRNA modification and the underlying molecular mechanisms of m^6^A regulatory proteins in various cancers (Fig. 3 and Table 1).

**Table 1:** Cellular effects of m^6^A mRNA modification in cancer.

	Positive regulation of m^6^A in cancer			Negative regulation of m^6^A in cancer		
	Molecular function	Target	Reference	Molecular function	Target	Reference
Lung cancer	Translation	A subset of mRNAs	^21,22^	mRNA level change	A subset of mRNAs	^68^
		*EGFR, TAZ, MAPKAPK2, DNMT3A* mRNA	^67^			
	Protein level change	BAX, BCL-2	^66^	mRNA stabilization	*MZF1* mRNA	^69^
Acute myeloid leukemia	Translation	A subset of mRNAs	^18^	mRNA stabilization	*ASB2*, *RARA* mRNA	^19^
		*MYC*, *BCL2*, *PTEN* mRNA	^71^			
	Translation	*MYB, MYC* mRNA	^73^	mRNA decay	A subset of mRNAs	^72^
	mRNA stabilization		^73^			
Hepatocellular carcinoma	mRNA level change	*SOCS2* mRNA	^75^	NR	NR	
	mRNA decay	*EGFR* mRNA	^76^			
	mRNA level change	*SON*, *SREBBP* mRNA	^77^			
Breast cancer	mRNA level change	*HBXIP* mRNA	^79^	mRNA level change	*NANOG*, *KFL4* mRNA	^80,81^
Gastric cancer	mRNA stabilization	*SEC62* mRNA	^83^	Unknown	Unknown	^85,86^
		*HDGF* mRNA	^84^			
Bladder cancer	mRNA level change	*AFF4*, *MYC* mRNA	^20^	NR	NR	
	Protein level change	AFF4, MYC, IKBKB, RELA	^20^			
	Translation	*ITGA6* mRNA	^87^			
Glioblastoma	mRNA stabilization	*SOX2* mRNA	^92^	mRNA level change	Nascent *FOXM1* transcript	^89^
				mRNA level change	A subset of mRNAs	^91^
Colorectal cancer	mRNA stabilization	*SOX2* mRNA	^93^	NR	NR	
Renal cell carcinoma	NR	NR		Unknown	Unknown	^95^
Endometerial Cancer	NR	NR		Translation	*PHLPP2* mRNA	^96^
				mRNA decay	*PRR5*, *PRR5L*, *mTOR* mRNA	^96^
Cervical cancer	NR	NR		Unknown	Unknown	^97^
Pancreatic cancer	NR	NR		Protein level change	YAP	^98^

Some studies did not identify molecular mechanisms or targets, but only measured m^6^A levels and their effects on cancer, which are marked as “unknown”. “Translation” indicates the m^6^A-mediated translation enhancement. “Protein level change” and “mRNA level change” indicate their steady-state levels without specifying the translation efficiency or mRNA stability.

*NR* not reported.

### Lung cancer

Lung cancer causes the greatest number of cancer-related deaths worldwide. There are two main histological types of lung cancer: small-cell lung cancer and non-small-cell lung cancer (NSCLC). Approximately 85% are classified as NSCLC, which statistically shows just a 15.9% 5-year survival rate^64,65^. Nevertheless, therapeutic efforts have improved only slightly over the last few decades. Therefore, it is urgent to explore new treatments and deepen our understanding of the underlying mechanisms of lung cancer occurrence and development. The relevance of m^6^A modification in lung cancer has been extensively studied, and several lines of evidence show that METTL3 is highly expressed in NSCLC cells and is associated with cell proliferation, invasion, and viability^21,22,66–68^. Two recent studies from the same group revealed intriguing effects of METTL3 in lung cancer progression. These studies showed that cytoplasm-localized METTL3 functions as an m^6^A reader protein that enhances translation of a large subset of oncogenic mRNAs without affecting mRNA abundance^21,22^. Mechanistically, the 3′ UTR near the stop codon-bound METTL3 directly interacts with eIF3h. This interaction mediates mRNA looping to facilitate the recycling of ribosomes at the termination codon in a similar way to canonical eukaryotic mRNA looping mediated by the interactions between eIF4E (a cap-binding protein), eIF4G (a translation initiation factor), and PABP (a poly(A)-binding protein)^21,22^. Indeed, ectopic expression of METTL3, but not a mutant that fails to interact with eIF3h, promotes cell proliferation, invasion, and oncogenic transformation^21^. Other studies have shown that *METTL3* mRNA can be targeted by microRNAs (miRNAs)^66,67^. Exogenously expressed miR-600 targets the 3′ UTR of *METTL3* mRNA, resulting in the inhibition of METTL3 expression^66^. Depletion of METTL3 inhibits the survival and proliferation of A549 and H1299 cells and leads to increased levels of the pro-apoptotic regulator BAX and decreased levels of the anti-apoptotic regulator BCL-2, suggesting that the altered expression ratio of BAX/BCL-2 triggers the mitochondrial apoptotic pathway^66^. In addition, knocking down METTL3 decreases the phosphorylation of AKT, thus affecting cell growth and apoptosis via the alteration of the PI3K/AKT/mTOR pathway^66^. Another miRNA, miR-33a, has also been shown to reduce METTL3 expression and, as a result, inhibits NSCLC cell proliferation^67^. On the other hand, METTL3 is SUMOylated by small ubiquitin-related modifier 1 (SUMO1), which modifies METTL3 at lysine residues and represses its methyltransferase activity without altering its stability, localization, or interaction with two other writer proteins, METTL14 and WTAP^68^. The SUMOylation of METTL3 reduces m^6^A levels and subsequently changes the mRNA expression profiles, ultimately promoting the development of NSCLC^68^. Besides, the m^6^A demethylase FTO has also been shown to play a critical role in lung squamous cell carcinoma (LUSC), one of the most common NSCLCs. FTO knockdown effectively inhibits cell proliferation and invasion while promoting apoptosis of L78 and NCI-H520 cells^69^. In contrast, overexpression of FTO, but not its mutant form, facilitates the acquisition of malignant phenotypes^69^. Mechanistically, FTO increases the stability of myeloid zinc finger 1 (*MZF1*) mRNA by reducing its m^6^A level, leading to high levels of protein expression, which has an oncogenic function^69^. MZF1 is a member of the SCAN-zinc finger transcription factor family, which contributes to cell proliferation, migration, and metastasis through the regulation of diverse target genes.

### Acute myeloid leukemia (AML)

AML is one of the most prevalent hematopoietic malignancies. It is often derived from genetic mutations and aberrant regulation of epigenetic modification, including DNA methylation and histone modification^70^. Recently, many studies have pointed to m^6^A mRNA modification as a new role for a gene expression regulator associated with AML^18,19,71,72^. As previously described, promoter-bound METTL3 induces m^6^A modification within coding regions of a subset of nascent transcripts independent of METTL14^18^. In this way, the genes necessary for AML growth enhance their translation efficiency by relieving ribosome stalling at the GAN (GAG, GAT, GAC, and GAA) codons during translation elongation^18^. Another study revealed that increased expression levels of METTL3 promote the translation of MYC proto-oncogene (*c-MYC*), B-cell lymphoma 2 (*BCL2*), and phosphatase and tensin homolog (*PTEN*) mRNAs by increasing the levels of m^6^A modification, thereby altering phosphoinositide 3-kinase (PI3K) and protein kinase B (PKB, also known as AKT) signaling, an intracellular signaling pathway important in regulating the cell cycle, to control cell differentiation and self-renewal^71^. METTL14 was also shown to function in a similar way by promoting translation of its target mRNAs, the proto-oncogenes *MYB* and *MYC*, through m^6^A modifications, which in turn leads to block the myeloid differentiation^73^. Notably, in addition to m^6^A writers, differentially expressed eraser or reader proteins seem to contribute to various AML subtypes through the modulation of m^6^A modification in a target mRNA-specific manner. Elevated expression of FTO enhances cell transformation and leukemogenesis by downregulating both the mRNA and protein expression of targets, such as *ASB2* and *RARA* mRNAs, by reducing the m^6^A levels in their UTRs^19^. On the other hand, YTHDF2 overexpression plays a crucial role in disease initiation and propagation in human and mouse AML by destabilizing a subset of mRNAs, including tumor necrosis factor receptor *TNFRSF2* mRNA^72^.

### Hepatocellular carcinoma (HCC)

HCC is a major type of primary liver cancer and is a highly progressive malignant tumor associated with a low survival rate^74^. It was recently reported that METTL3 levels are increased in human HCC, leading to increased m^6^A modification of the tumor suppressor *SOCS2* mRNA^75^. Increased levels of m^6^A in *SOCS2* mRNA can be targeted by YTHDF2, leading to its rapid degradation, which is associated with the efficient proliferation of HCC cells^75^. Besides, overexpression of YTHDF2 has been shown to suppress cell proliferation and tumor growth in HCC cells^76^. Mechanistically, the m^6^A-modified 3′ UTR of epidermal growth factor receptor (*EGFR*) mRNA is recognized by YTHDF2 and undergoes degradation, which in turn impairs mitogen-activated protein kinase kinase (MEK) and extracellular signal-regulated kinases (ERK)^76^. Similarly, another report showed that *YTHDF2* mRNA can be targeted by miR145, leading to an increase in overall m^6^A levels in HCC cells, which is associated with HCC malignancy^77^.

### Breast cancer (BrC)

Of all malignant tumors in women, BrC is highly metastatic and has the highest cancer-related mortality^78^. One interesting report suggested a potential positive feedback loop between mammalian hepatitis B X-interacting protein (HBXIP) and METTL3^79^. High expression levels of HBXIP elevate METTL3 expression through the suppression of let-7g, and increased METTL3 upregulates HBXIP expression through m^6^A modifications of mRNA. This positive feedback loop leads to the acceleration of cell proliferation in BrC. On the other hand, a decrease in m^6^A modification also promotes BrC tumorigenesis. In BrC stem cells, hypoxic stress induces overexpression of ALKBH5 and/or ZNF217, leading to inhibition of the methylation of pluripotency markers *NANOG* and *KLF4* mRNAs^80,81^. Increasing the expression of *NANOG* and *KLF4* mRNA by inhibiting m^6^A modification promotes the specification of BrC stem cells^80,81^. Another report also showed that m^6^A levels increased by METTL14 overexpression or ALKBH5 knockdown inhibited BrC growth and metastasis^82^.

### Gastric cancer (GC)

GC is a prevalent tumor occurring in the digestive system. One clear mechanism showed that the preprotein translocation factor *SEC62* mRNA can undergo m^6^A modification by METTL3^83^. In turn, IGF2BP1 recognizes m^6^A and facilitates the stabilization of *SEC62* mRNA. Moreover, miR4429 has been suggested to target METTL3 and prevent the m^6^A modification of *SEC62* mRNA, thus destabilizing *SEC62* mRNA^83^. Downregulated SEC62 inhibits GC cell proliferation and promotes apoptosis^83^. Another report showed that METTL3 transcription is elevated in GC by a histone acetyltransferase, P300, which mediates H3K27 acetylation at the METTL3 promoter region, which in turn induces the methylation of hepatoma-derived growth factor (*HDGF*) mRNA^84^. The methylated *HDGF* mRNA is then recognized and stabilized by IGF2BP3. Overexpressed HDGF protein can be secreted and promotes tumor angiogenesis, while nuclear HDGF stimulates the expression of glucose transporter type 4 (*GLUT-4*) and enolase 2 (*ENO2*) mRNAs, resulting in increased levels of glycolysis and subsequently causing tumor growth and liver metastasis^84^. On the other hand, it has been suggested that FTO and ALKBH1 play crucial roles in GC progression and metastasis, although the relevance of m^6^A in these processes is unclear^85^. It has been shown statistically that lower ALKBH1 protein expression correlates with larger tumor size, while lower FTO protein expression is associated with shorter overall survival in patients with GC^85^. Another report revealed that the downregulation of m^6^A modification by METTL14 knockdown leads to the acquisition of oncogenic phenotypes through the alteration of Wnt and PI3K-AKT signaling pathways, although the exact upstream regulatory mechanism is unclear^86^.

### Bladder cancer (BlC)

BlC is the most prevalent urogenital cancer. Recent studies suggest that increased levels of m^6^A modification are correlated with BlC^20,87^. One study identified the mRNAs of AF4/FMR2 family member 4 (*AFF4*), two key regulators of the NF-κB pathway (*IKBKB* and *RELA*), and *MYC* as direct METTL3 targets for m^6^A modification^20^. METTL3 depletion led to a reduction in *AFF4* and *MYC* mRNA and protein expression, while only the protein expression was reduced for IKBKB and RELA. METTL3 downregulation in BlC drastically reduced cell proliferation, invasion, and survival in vitro and tumorigenicity in vivo^20^. Considering the results indicating that (1) MYC is a well-known oncogene that triggers the expression of target genes to benefit cell proliferation, cell survival, and stemness maintenance and (2) AFF4 and NF-κB are known to regulate MYC expression, through which NF-κB signaling enhances the proliferation and survival of cancer cells during the development and recurrence of BlC, it can be speculated that m^6^A modification by METTL3 affects the AFF4/NF-κB/MYC signaling network to regulate BlC progression^20^. In addition, upregulated METTL3 promotes the translation of integrin alpha-6 (*ITGA6*) mRNA via the recognition of m^6^A in the 3′ UTR by the m^6^A reader proteins YTHDF1 and YTHDF3^87^. As a result, the upregulated ITGA6 protein promotes BlC cell adhesion, migration, and invasion, similar to multiple other types of cancer, in which ITGA6 overexpression promotes tumorigenesis and metastasis^87^.

### Glioblastoma (GBM)

GBM is a primary malignant brain tumor prevalent in adults^88^. GBMs have heterogeneous characteristics and contain cells with stem-like properties^89^. These self-renewing GBM stem-like cells (GSCs) contribute to tumor initiation and therapeutic resistance^90^. Intriguingly, the expression levels of both METTL3 and ALKBH5 are elevated in GSCs, with opposite results on m^6^A-mediated tumor formation in a target-specific manner^89,91,92^. High METTL3 expression levels exhibit oncogenic function through efficient m^6^A modification in the 3′ UTR of sex-determining region Y (SRY)-box 2 (*SOX2*) mRNA, which is stabilized by binding of HuR^92^. Silencing METTL3 expression reduces SOX2 expression and, as a result, inhibits GBM tumor growth and prolongs the survival of mice^92^. In contrast, ALKBH5 is highly expressed in GSCs and demethylates *FOXM1* nascent transcripts, leading to FOXM1 overexpression, stem-like cell proliferation, and tumorigenesis^89^. The elevated levels of the transcription factor FOXM1 play critical roles in regulating GSC proliferation, self-renewal, and tumorigenicity^89^. Similarly, another study suggested a tumor-suppressive function for the m^6^A modification in GSCs^91^. Reduction of m^6^A modification by the depletion of METTL3 or METTL14 or the chemical inhibition of FTO upregulates the mRNA expression of critical oncogenes such as *ADAM19*, *EPHA3*, and *KLF4* and downregulates the mRNA expression of many tumor suppressors, including *CDKN2A*, *BRCA2*, and *TP53I11* mRNAs, resulting in overall enhanced GBM stem cell growth, self-renewal, and tumorigenesis^91^.

### Colorectal cancer (CrC)

In CrC, METTL3 and YTHDF1 expression is significantly upregulated^93,94^. High levels of METTL3 expression have been shown to significantly upregulate m^6^A methylation in the coding sequences of *SOX2* mRNA, a well-known CrC marker that is involved in maintaining the properties of tumor-initiating cells^93^. Methylated *SOX2* mRNA is subsequently recognized by IGF2BP2, preventing mRNA degradation. Indeed, knocking down METTL3 reduces the SOX2 expression level, inhibiting CrC development and metastasis^93^. On the other hand, c-MYC has been suggested to promote YTHDF1 transcription^94^. A statistical analysis suggests that patients with high YTHDF1 expression have significantly poorer overall survival^94^. Moreover, knocking down YTHDF1 results in the inhibition of cell proliferation and sensitization of cells to anticancer drugs such as fluorouracil and oxaliplatin^94^.

### Other cancers

Similar to the cancers discussed above, modulation of m^6^A modification plays a critical role in renal cell carcinoma, endometrial cancer, and cervical cancer^95–97^. In renal cell carcinoma, depletion of METTL3 promotes cell proliferation, cell invasion, and migration, and induces G0/G1 arrest^95^. Conversely, upregulation of METTL3 results in significant suppression of tumor growth^95^. Moreover, knocking down METTL3 promotes the acquisition of an epithelial phenotype and represses the manifestation of a mesenchymal phenotype, while overexpression of METTL3 reverses epithelial–mesenchymal transition progression^95^. Furthermore, the observation that increased phosphorylation levels of PI3K/AKT/mTOR due to METTL3 knockdown suggests that these METTL3-mediated pathways may also be involved in renal cell carcinoma progression^95^. A report revealed that METTL14 is frequently mutated and METTL3 expression is significantly reduced in endometrial cancer^96^. Mechanistically, m^6^A mRNA modification affects the YTHDF1-dependent translation enhancement of the negative AKT regulator PHLPP2 and YTHDF2-dependent destabilization of the mRNAs of positive AKT regulators *PRR5*, *PRR5L*, and *mTOR*. Thus, either METTL14 mutation or decreased METTL3 expression leads to m^6^A reduction in these target mRNAs and, as a result, promotes cell proliferation and tumorigenicity of endometrial cancer through AKT activation^96^. In cervical cancer, downregulation of m^6^A modification enhances cell proliferation, while upregulation inhibits tumor development^97^. However, the exact mechanism remains unknown. Last, YTHDF2 is upregulated in pancreatic cancer and has two roles in cancer development: 1) YTHDF2 promotes cell proliferation, since it was observed that knocking down YTHDF2 results in the activation of the AKT/GSK3β/Cyclin D1 pathway, leading to G1 arrest, and 2) the YTHDF2-mediated decay of yes-associated protein (YAP) may influence the epithelial–mesenchymal transition, since overexpression of YAP results in decreased expression of epithelial markers and increases in mesenchymal markers^98^.

## Concluding remarks and future perspectives

Considering the increasing number of studies revealing that m^6^A modification plays a critical role in almost all stages of mRNA metabolism^10,48,56,62^, we can easily speculate that aberrant regulation of these modifications affects many cellular phenotypes. Nevertheless, the molecular mechanisms and cellular effects of m^6^A mRNA modifications are not yet fully understood, since they do not always function in the same way. For instance, although it is well known that the m^6^A modification sites in mRNAs are mainly enriched in the 3′ UTR near the stop codon^3,12,42^, several recent findings showed that cotranscriptional methylation occurs in coding sequences (Fig. 1)^18,27^. In addition, it is still unclear why some mRNAs are not methylated. Considering that the m^6^A modification is reversible, the demethylases FTO and/or ALKBH5 may play critical roles in balancing the methylation of specific mRNAs in a cell type-dependent manner.

In recent years, m^6^A modification studies in various cancers have been conducted. Remarkably, an increasing number of studies have revealed that altered expression levels of m^6^A methyltransferases, demethylases, and reader proteins aberrantly regulate m^6^A modification on target mRNAs, resulting in abnormal expression of cancer-associated genes. In particular, increased methyltransferase expression levels were detected in most cancers, suggesting that higher m^6^A modification levels are closely related to tumorigenesis. However, the molecular functions and cellular consequences of m^6^A modification differed in each study, depending on the degree of methylation in the specific target mRNAs (Table 1). For instance, increased levels of m^6^A modification by higher levels of METTL3 or METTL14 expression promoted the translation or stabilization of *c-MYC*, *BCL2*, *PTEN*, or *MYB* mRNAs in AML^71,73^. In contrast, FTO also showed an elevated level of expression, which downregulated both the translation and abundance of *ASB2* and *RARA* mRNAs through demethylation^19^. Taken together, the coordinated functions of methylation and demethylation of specific targets seem to be critical for tumorigenesis.

Interest in m^6^A modification resurged quite recently. To date, most of the m^6^A studies in cancer have been demonstrated based on the discovery of the m^6^A modification itself rather than the underlying mechanisms with reader proteins (Fig. 3 and Table 1) because efforts to define the molecular mechanism and the biological relevance have been carried out in parallel. To date, only a single m^6^A reader-dependent molecular mechanism has been demonstrated in most cancer types (Fig. 3). In addition, cancer-related studies on other outcomes of m^6^A-dependent mRNA regulation, such as pre-mRNA splicing or mRNA export, remain insufficient. Considering that multiple reader proteins recognize m^6^A, it might be possible to crosstalk between readers on a single or a multiple m^6^A modification in an mRNA for the tight gene expression regulation. Therefore, to develop novel tumor therapies based on the regulation of m^6^A modifications, more thorough mechanistic and functional studies are required for each cancer type.

## References

[1] Heard E, Martienssen RA (2014). Transgenerational epigenetic inheritance: myths and mechanisms. Cell.

[2] Esteller M, Pandolfi PP (2017). The epitranscriptome of noncoding RNAs in cancer. Cancer Discov..

[3] Meyer KD (2012). Comprehensive analysis of mRNA methylation reveals enrichment in 3’ UTRs and near stop codons. Cell.

[4] Helm M, Motorin Y (2017). Detecting RNA modifications in the epitranscriptome: predict and validate. Nat. Rev. Genet..

[5] Nachtergaele S, He C (2018). Chemical modifications in the life of an mRNA transcript. Annu. Rev. Genet..

[6] Meyer KD, Jaffrey SR (2014). The dynamic epitranscriptome: N6-methyladenosine and gene expression control. Nat. Rev. Mol. Cell Biol..

[7] Wang X (2014). N6-methyladenosine-dependent regulation of messenger RNA stability. Nature.

[8] Kasowitz SD (2018). Nuclear m6A reader YTHDC1 regulates alternative polyadenylation and splicing during mouse oocyte development. PLoS Genet..

[9] Liu N (2015). N(6)-methyladenosine-dependent RNA structural switches regulate RNA-protein interactions. Nature.

[10] Roundtree IA (2017). YTHDC1 mediates nuclear export of N(6)-methyladenosine methylated mRNAs. Elife.

[11] Li A (2017). Cytoplasmic m(6)A reader YTHDF3 promotes mRNA translation. Cell Res..

[12] Dominissini D (2012). Topology of the human and mouse m6A RNA methylomes revealed by m6A-seq. Nature.

[13] Schwartz S (2013). High-resolution mapping reveals a conserved, widespread, dynamic mRNA methylation program in yeast meiosis. Cell.

[14] Clancy MJ, Shambaugh ME, Timpte CS, Bokar JA (2002). Induction of sporulation in Saccharomyces cerevisiae leads to the formation of N6-methyladenosine in mRNA: a potential mechanism for the activity of the IME4 gene. Nucleic Acids Res.

[15] Zhong S (2008). MTA is an Arabidopsis messenger RNA adenosine methylase and interacts with a homolog of a sex-specific splicing factor. Plant Cell.

[16] Lin Z (2017). Mettl3-/Mettl14-mediated mRNA N(6)-methyladenosine modulates murine spermatogenesis. Cell Res.

[17] Wang Y (2018). Publisher correction: N(6)-methyladenosine RNA modification regulates embryonic neural stem cell self-renewal through histone modifications. Nat. Neurosci..

[18] Barbieri I (2017). Promoter-bound METTL3 maintains myeloid leukaemia by m(6)A-dependent translation control. Nature.

[19] Li Z (2017). FTO plays an oncogenic role in acute myeloid leukemia as a N(6)-methyladenosine RNA demethylase. Cancer Cell.

[20] Cheng M (2019). The m(6)A methyltransferase METTL3 promotes bladder cancer progression via AFF4/NF-kappaB/MYC signaling network. Oncogene.

[21] Choe J (2018). mRNA circularization by METTL3-eIF3h enhances translation and promotes oncogenesis. Nature.

[22] Lin S, Choe J, Du P, Triboulet R, Gregory RI (2016). The m(6)A methyltransferase METTL3 promotes translation in human cancer cells. Mol. Cell.

[23] Wu R, Jiang D, Wang Y, Wang X (2016). N (6)-Methyladenosine (m(6)A) methylation in mRNA with a dynamic and reversible epigenetic modification. Mol. Biotechnol..

[24] Wang P, Doxtader KA, Nam Y (2016). Structural basis for cooperative function of Mettl3 and Mettl14 methyltransferases. Mol. Cell.

[25] Jia G, Fu Y, He C (2013). Reversible RNA adenosine methylation in biological regulation. Trends Genet.

[26] Knuckles P (2017). RNA fate determination through cotranscriptional adenosine methylation and microprocessor binding. Nat. Struct. Mol. Biol..

[27] Slobodin B (2017). Transcription impacts the efficiency of mRNA translation via co-transcriptional N6-adenosine Methylation. Cell.

[28] Knuckles P (2018). Zc3h13/Flacc is required for adenosine methylation by bridging the mRNA-binding factor Rbm15/Spenito to the m(6)A machinery component Wtap/Fl(2)d. Genes Dev..

[29] Wen J (2018). Zc3h13 regulates nuclear RNA m(6)A methylation and mouse embryonic stem cell self-renewal. Mol. Cell.

[30] Ping XL (2014). Mammalian WTAP is a regulatory subunit of the RNA N6-methyladenosine methyltransferase. Cell Res.

[31] Patil DP (2016). m(6)A RNA methylation promotes XIST-mediated transcriptional repression. Nature.

[32] Yue Y (2018). VIRMA mediates preferential m(6)A mRNA methylation in 3’UTR and near stop codon and associates with alternative polyadenylation. Cell Discov..

[33] Pendleton KE (2017). The U6 snRNA m(6)A methyltransferase METTL16 regulates SAM synthetase intron retention. Cell.

[34] Warda AS (2017). Human METTL16 is a N(6)-methyladenosine (m(6)A) methyltransferase that targets pre-mRNAs and various non-coding RNAs. EMBO Rep..

[35] Shima H (2017). S-Adenosylmethionine synthesis is regulated by selective N(6)-adenosine methylation and mRNA degradation involving METTL16 and YTHDC1. Cell Rep..

[36] Jia G (2011). N6-methyladenosine in nuclear RNA is a major substrate of the obesity-associated FTO. Nat. Chem. Biol..

[37] Zheng G (2013). ALKBH5 is a mammalian RNA demethylase that impacts RNA metabolism and mouse fertility. Mol. Cell.

[38] Dina C (2007). Variation in FTO contributes to childhood obesity and severe adult obesity. Nat. Genet.

[39] Fu Y (2013). FTO-mediated formation of N6-hydroxymethyladenosine and N6-formyladenosine in mammalian RNA. Nat. Commun..

[40] Mauer J (2017). Reversible methylation of m(6)Am in the 5’ cap controls mRNA stability. Nature.

[41] Wei J (2018). Differential m(6)A, m(6)Am, and m(1)A demethylation mediated by FTO in the cell nucleus and cytoplasm. Mol. Cell.

[42] Linder B (2015). Single-nucleotide-resolution mapping of m6A and m6Am throughout the transcriptome. Nat. Methods.

[43] Lence T (2016). m(6)A modulates neuronal functions and sex determination in Drosophila. Nature.

[44] Haussmann IU (2016). m(6)A potentiates Sxl alternative pre-mRNA splicing for robust Drosophila sex determination. Nature.

[45] Zhao X (2014). FTO-dependent demethylation of N6-methyladenosine regulates mRNA splicing and is required for adipogenesis. Cell Res..

[46] Tang C (2018). ALKBH5-dependent m6A demethylation controls splicing and stability of long 3’-UTR mRNAs in male germ cells. Proc. Natl Acad. Sci. USA.

[47] Liu N (2017). N6-methyladenosine alters RNA structure to regulate binding of a low-complexity protein. Nucleic Acids Res.

[48] Xiao W (2016). Nuclear m(6)A reader YTHDC1 regulates mRNA splicing. Mol. Cell.

[49] Jacobs E, Mills JD, Janitz M (2012). The role of RNA structure in posttranscriptional regulation of gene expression. J. Genet Genomics.

[50] Roost C (2015). Structure and thermodynamics of N6-methyladenosine in RNA: a spring-loaded base modification. J. Am. Chem. Soc..

[51] Wang X (2015). N(6)-methyladenosine modulates messenger RNA translation efficiency. Cell.

[52] Shi H (2017). YTHDF3 facilitates translation and decay of N(6)-methyladenosine-modified RNA. Cell Res.

[53] Hsu PJ (2017). Ythdc2 is an N(6)-methyladenosine binding protein that regulates mammalian spermatogenesis. Cell Res.

[54] Wojtas MN (2017). Regulation of m(6)A transcripts by the 3’->5’ RNA helicase YTHDC2 is essential for a successful meiotic program in the mammalian germline. Mol. Cell.

[55] Park OH (2019). Endoribonucleolytic cleavage of m(6)A-containing RNAs by RNase P/MRP complex. Mol. Cell.

[56] Du H (2016). YTHDF2 destabilizes m(6)A-containing RNA through direct recruitment of the CCR4-NOT deadenylase complex. Nat. Commun..

[57] Zhou J (2015). Dynamic m(6)A mRNA methylation directs translational control of heat shock response. Nature.

[58] Meyer KD (2015). 5’ UTR m(6)A promotes cap-independent translation. Cell.

[59] Ries RJ (2019). m(6)A enhances the phase separation potential of mRNA. Nature.

[60] Zhang F (2018). Fragile X mental retardation protein modulates the stability of its m6A-marked messenger RNA targets. Hum. Mol. Genet..

[61] Edupuganti RR (2017). N(6)-methyladenosine (m(6)A) recruits and repels proteins to regulate mRNA homeostasis. Nat. Struct. Mol. Biol..

[62] Huang H (2018). Recognition of RNA N(6)-methyladenosine by IGF2BP proteins enhances mRNA stability and translation. Nat. Cell Biol..

[63] Wang Y (2014). N6-methyladenosine modification destabilizes developmental regulators in embryonic stem cells. Nat. Cell Biol..

[64] Chen Z, Fillmore CM, Hammerman PS, Kim CF, Wong KK (2014). Non-small-cell lung cancers: a heterogeneous set of diseases. Nat. Rev. Cancer.

[65] Molina JR, Yang P, Cassivi SD, Schild SE, Adjei AA (2008). Non-small cell lung cancer: epidemiology, risk factors, treatment, and survivorship. Mayo Clin. Proc..

[66] Wei W, Huo B, Shi X (2019). miR-600 inhibits lung cancer via downregulating the expression of METTL3. Cancer Manag. Res..

[67] Du M (2017). MiR-33a suppresses proliferation of NSCLC cells via targeting METTL3 mRNA. Biochem Biophys. Res Commun..

[68] Du Y (2018). SUMOylation of the m6A-RNA methyltransferase METTL3 modulates its function. Nucleic Acids Res.

[69] Liu J (2018). m(6)A demethylase FTO facilitates tumor progression in lung squamous cell carcinoma by regulating MZF1 expression. Biochem. Biophys. Res. Commun..

[70] Chen J, Odenike O, Rowley JD (2010). Leukaemogenesis: more than mutant genes. Nat. Rev. Cancer.

[71] Vu LP (2017). The N(6)-methyladenosine (m(6)A)-forming enzyme METTL3 controls myeloid differentiation of normal hematopoietic and leukemia cells. Nat. Med.

[72] Paris J (2019). Targeting the RNA m(6)A reader ythdf2 selectively compromises cancer stem cells in acute myeloid leukemia. Cell Stem Cell.

[73] Weng H (2018). METTL14 inhibits hematopoietic stem/progenitor differentiation and promotes leukemogenesis via mRNA m(6)A modification. Cell Stem Cell.

[74] Sia D, Villanueva A, Friedman SL, Llovet JM (2017). Liver cancer cell of origin, molecular class, and effects on patient prognosis. Gastroenterology.

[75] Chen M (2018). RNA N6-methyladenosine methyltransferase-like 3 promotes liver cancer progression through YTHDF2-dependent posttranscriptional silencing of SOCS2. Hepatology.

[76] Zhong L (2019). YTHDF2 suppresses cell proliferation and growth via destabilizing the EGFR mRNA in hepatocellular carcinoma. Cancer Lett..

[77] Yang Z (2017). MicroRNA-145 modulates N(6)-methyladenosine levels by targeting the 3’-untranslated mRNA region of the N(6)-methyladenosine binding YTH domain family 2 protein. J. Biol. Chem..

[78] DeSantis CE, Ma J, Goding Sauer A, Newman LA, Jemal A (2017). Breast cancer statistics, 2017, racial disparity in mortality by state. CA Cancer J. Clin..

[79] Cai X (2018). HBXIP-elevated methyltransferase METTL3 promotes the progression of breast cancer via inhibiting tumor suppressor let-7g. Cancer Lett..

[80] Zhang C (2016). Hypoxia-inducible factors regulate pluripotency factor expression by ZNF217- and ALKBH5-mediated modulation of RNA methylation in breast cancer cells. Oncotarget.

[81] Zhang C (2016). Hypoxia induces the breast cancer stem cell phenotype by HIF-dependent and ALKBH5-mediated m(6)A-demethylation of NANOG mRNA. Proc. Natl Acad. Sci. USA.

[82] Wu L, Wu D, Ning J, Liu W, Zhang D (2019). Changes of N6-methyladenosine modulators promote breast cancer progression. BMC Cancer.

[83] He H, Wu W, Sun Z, Chai L (2019). MiR-4429 prevented gastric cancer progression through targeting METTL3 to inhibit m(6)A-caused stabilization of SEC62. Biochem. Biophys. Res Commun..

[84] Wang, Q. et al. METTL3-mediated m(6)A modification of HDGF mRNA promotes gastric cancer progression and has prognostic significance. *Gut* (2019). [Epub ahead of print] 10.1136/gutjnl-2019-319639 31582403

[85] Li Y (2019). Expression of demethylase genes, FTO and ALKBH1, is associated with prognosis of gastric cancer. Dig. Dis. Sci..

[86] Zhang C (2019). Reduced m6A modification predicts malignant phenotypes and augmented Wnt/PI3K-Akt signaling in gastric cancer. Cancer Med..

[87] Jin H (2019). N(6)-methyladenosine modification of ITGA6 mRNA promotes the development and progression of bladder cancer. EBioMedicine.

[88] Thakkar JP (2014). Epidemiologic and molecular prognostic review of glioblastoma. Cancer Epidemiol. Biomark. Prev..

[89] Zhang S (2017). m(6)A demethylase ALKBH5 maintains tumorigenicity of glioblastoma stem-like cells by sustaining FOXM1 expression and cell proliferation program. Cancer Cell.

[90] Lathia JD, Mack SC, Mulkearns-Hubert EE, Valentim CL, Rich JN (2015). Cancer stem cells in glioblastoma. Genes Dev..

[91] Cui Q (2017). m(6)A RNA methylation regulates the self-renewal and tumorigenesis of glioblastoma stem cells. Cell Rep..

[92] Visvanathan A (2018). Essential role of METTL3-mediated m(6)A modification in glioma stem-like cells maintenance and radioresistance. Oncogene.

[93] Li T (2019). METTL3 facilitates tumor progression via an m(6)A-IGF2BP2-dependent mechanism in colorectal carcinoma. Mol. Cancer.

[94] Nishizawa Y (2017). Oncogene c-Myc promotes epitranscriptome m6A reader YTHDF1 expression in colorectal cancer. Oncotarget.

[95] Li X (2017). The M6A methyltransferase METTL3: acting as a tumor suppressor in renal cell carcinoma. Oncotarget.

[96] Liu J (2018). m(6)A mRNA methylation regulates AKT activity to promote the proliferation and tumorigenicity of endometrial cancer. Nat. Cell Biol..

[97] Wang X (2017). Reduced m6A mRNA methylation is correlated with the progression of human cervical cancer. Oncotarget.

[98] Chen J (2017). YTH domain family 2 orchestrates epithelial-mesenchymal transition/proliferation dichotomy in pancreatic cancer cells. Cell Cycle.

